# Improvement in functional and mental outcomes after resection rectopexy for obstructive defecation syndrome ODS

**DOI:** 10.1038/s41598-025-15037-1

**Published:** 2025-08-18

**Authors:** Claudia Rudroff, Joshy Madukkakuzhy, Jakob Otten, Julia Schroer, Dana Richards, Sebastian Ludwig, Michaela Henning

**Affiliations:** 1 Department of Urology and Uro-Oncology, Robot-assisted and Specialized Urologic Surgery, Division of Pelvic Floor Surgery, University of Cologne, Faculty of Medicine and University Hospital, Kerpener Strasse 62, 50937 Cologne, Germany; 2 Department of General and Visceral Surgery, Evangelisches Klinikum Koeln Weyertal, Academic Teaching Hospital of the University of Cologne, Cologne, Germany; 3 Department of Oral, Maxillofacial and Plastic Surgery, University Hospital Bonn, Bonn, Germany; 4 University of Cologne, Cologne, Germany; 5 Department for Gynecology, Division of Urogynecology and Pelvic Reconstructive Surgery, University of Cologne, Medical Faculty and University Hospital, Cologne, Germany; 6 Department for Psychosomatics and Psychotherapy, University of Cologne, Medical Faculty and University Hospital, Cologne, Germany

**Keywords:** Obstructed defecation syndrome (ODS), Depression, Anxiety, Interdisciplinary surgery, Mental outcome, Mental burden, Functional gastrointestinal disorders, Outcomes research, Human behaviour

## Abstract

Obstructive defecation syndrome (ODS) is a condition that causes straining and may require manual evacuation and patients’ thoughts circle around defecation impairing their quality of life. Mental comorbidity-related findings suggest a mental burden in ODS patients. In an observational cohort design, this study investigated the relationship between the mental distress and their clinical symptom severity for a group of patients with ODS who underwent surgical treatment. This study included 108 consecutive patients with ODS who were scheduled for laparoscopic resection rectopexy combined with pelvic floor repair, if indicated. Clinical symptom severity (Altomare score, rectal toxicity score, and Wexner incontinence score) and mental health scores (Generalized Anxiety Disorder-7 [GAD-7] and Personal Health Questionnaire-9 [PHQ-9]) were assessed before and 6 months after surgery. Before surgery, 82.5% of patients had at least mild depressive symptoms (PHQ-9 score ≥ 5), and 55.6% of patients had at least mild anxiety (GAD-7 score ≥ 5). The severity of the mental health scores correlated with the clinical symptom severity. At the 6-month follow-up, the bowel function scores improved significantly. Depression symptoms improved, whereas only slight changes in anxiety symptoms were observed. The improvement in clinical symptom severity correlated with better mental score results, whereas the severity of the preoperative mental distress did not influence the surgical or follow-up outcomes. Patients with obstructive defecation syndrome (ODS) experience significant depression and anxiety that adversely affect their quality of life. Surgical improvement of bowel function reduces depressive symptoms and, to a lesser extent, anxiety symptoms. Early multidisciplinary intervention should be considered for effective management. Future studies should further investigate mental distress caused by ODS symptoms or other underlying psychiatric comorbidities.

## Introduction

Obstructive defecation syndrome (ODS) is characterized by an impaired defecation process due to functional muscular abnormalities, such as failure of pelvic floor muscle coordination or relaxation—as observed in defecation dyssynergia or anismus—or anatomical defects like rectocele (protrusion of the rectal wall), internal rectal prolapse (intussusception), or enterocele^1,2^ .

The sensation of obstruction at the level of the pelvic floor leads to excessive straining and may contribute to the development of pelvic organ prolapse, which often exacerbates the progression of ODS and is associated with chronic constipation in many cases^3^. Additionally, manual manipulation may be required to facilitate rectal evacuation. Multiple and often unsuccessful defecation attempts leave ODS patients with a sense of incomplete rectal emptying. Patients often experience significant frustration, with preoccupation on defecation adversely affecting their quality of life.

Approximately 10%–25% of the global population is affected by ODS, with the incidence increasing with age^4–7^. The incidence of ODS is predominantly in women, where it is often associated with pelvic organ prolapse (POP). However, men are also affected.

When conservative treatment options fail, surgery may be required to reconstruct the bowel and pelvic floor anatomy. Mental and psychological comorbidities have rarely been studied in this clinical setting. Existing studies have suggested a greater burden of anxiety and depression in patients with ODS than in other somatic patients^8,9^, with anxiety even being identified as a possible prognostic outcome parameter for surgery^10^.

This study examined the mental distress in a group of ODS patients who underwent surgery for this condition.

and correlated the findings with clinical symptom severity before and 6 months after the intervention.

## Patients and methods

### Aim, design, and setting of the study

This retrospective observational cohort study was performed at the Evangelisches Klinikum Koeln Weyertal, an academic teaching hospital associated with the University of Cologne, which accommodates a tertiary pelvic organ and bowel function center (POC) as part of the surgical department. Patients with obstructive defecation syndrome (ODS) frequently present to our department for multidisciplinary consultation and evaluation of surgical therapy options. All patients undergo a comprehensive diagnostic workup as described below. Subsequently, all cases are routinely presented at the Pelvic Organ Center (POC) board to discuss individualized treatment recommendations. This includes surgery for patients with anatomical defects and alternative options, such as enhanced conservative therapy, for those with solely functional muscular abnormalities. All consecutive patients scheduled for surgery were included in this study.

Between February 2020 and March 2023 altogether 108 consecutive patients were included in this study. The self-reporting clinical and mental score data were prospectively acquired as part of the standard operating procedure for all patients with functional bowel motility disorders. Informed consent for the planned procedure, data collection, and publication of the results was obtained from all patients before surgery. The study was approved by the local Ethics Committee (No: 74/2024) at the Aerztekammer Northrhine in Duesseldorf, Germany. The study was retrospectively registered with ClinicalTrials.gov: NCT06502184.

### Study endpoints

The primary study outcome parameter was mental health after surgery. The results were correlated with the clinical outcomes, which were measured based on bowel function scores. All scores were routinely assessed before surgery and at the 6-month postsurgical follow-up.

### Patient characteristics and clinical and mental scores

Data regarding patient characteristics, such as age, sex, body mass index (BMI), and comorbidities, as determined by the American Society of Anesthesiologists (ASA) score^11^, were collected.

Defecation symptoms were measured using the Altomare score, a validated ODS questionnaire with a maximum of 30 points^12^. Abdominal and bowel discomfort were assessed by the rectal toxicity score, a validated German questionnaire for patients after radiation for pelvic malignancies^13–15^. The score asks for bowel dysfunction symptoms, such as diarrhea, meteorism, bleeding, and abdominal pain during bowel movement and defecation and has a maximum of 32 points. The Wexner incontinence score is an established questionnaire for fecal incontinence symptoms, with a maximum of 20 points^16,17^. On all the questionnaires, higher scores indicate greater severity of symptoms with no cutoff levels defined.

Mental disorders were evaluated using the personal health questionnaire-9 (PHQ-9) and the generalized anxiety disorder questionnaire-7 (GAD-7). The PHQ-9 scores are divided into five subgroups of depression severity, namely, nonminimal (0–4 points), mild (5–9 points), moderate (10–14 points), moderately severe (15–19 points), and severe (20–27 points)^18^. The GAD-7 has four subgroups that differentiate anxiety into minimal (0–4 points), mild (5–9 points), moderate (10–14 points), and severe levels (15–21 points)^19^.

### Diagnostic work up

**Fig. 1:**
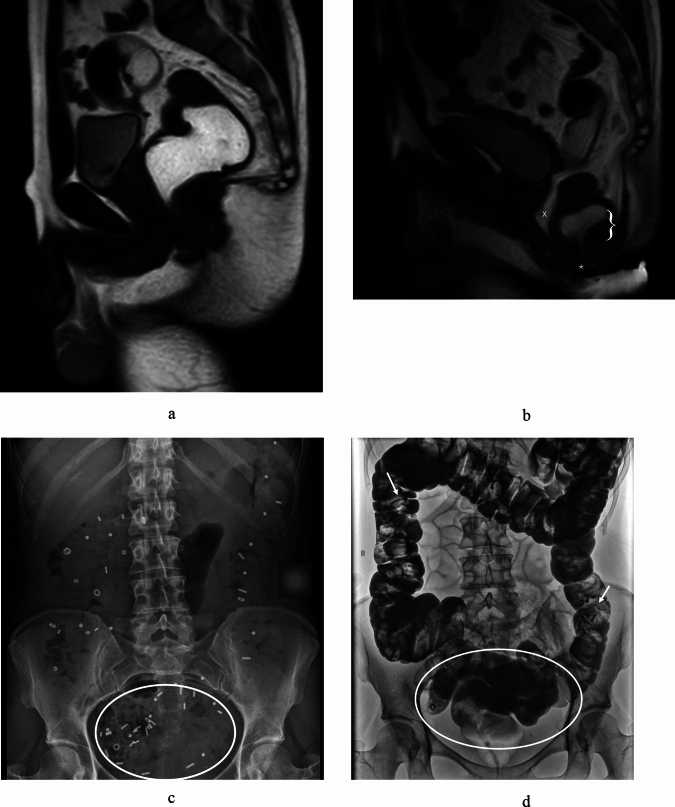
Representative radiologic images of the diagnostic work up for ODS. Magnetic resonance image defecography (MRI-D) in sagittal view of a male pelvis showing (**A**) the situation at rest and (**B**) at the end of defecation with a rectal prolapse (marked with *), the signs of the descending peritoneum as in a pelvic organ prolapse (marked with X), and intussusception of the distal rectum (marked with parenthesis “}”) .The X-ray of a colonic transit time (**C**) with distribution of the opaque markers in the ascending colon and the rectosigmoid (circled with a white ellipse). Image of the corresponding colon enema (**D**) showing an elongated rectosigmoid (circled white ellipse) and some residual markers from the colonic transit time examination 5 days and a bowel preparation later (white arrows).

All patients included in this study were examined by a trained colorectal surgeon and an uro-gynecologist to assess pelvic organ prolapse (POP) in case of female patients. A magnetic resonance image defecography (MRI-D) was routinely performed before surgery. The results from the clinical examination and the radiologic imaging served to define the anatomical defect causing the ODS, as illustrated in Fig. 1 below.

The colonic transit time analysis and a colon enema were added if patients complained about constipation^20,21^. To rule out other abdominal pathologies a computed tomography or a magnetic resonance image of the abdomen was performed in selected cases.

### Surgical procedure

A laparoscopic resection rectopexy was the standard procedure performed under general anesthesia with the patients in lithotomy position with a Trendelenburg tilt (head down).

**Fig. 2:**
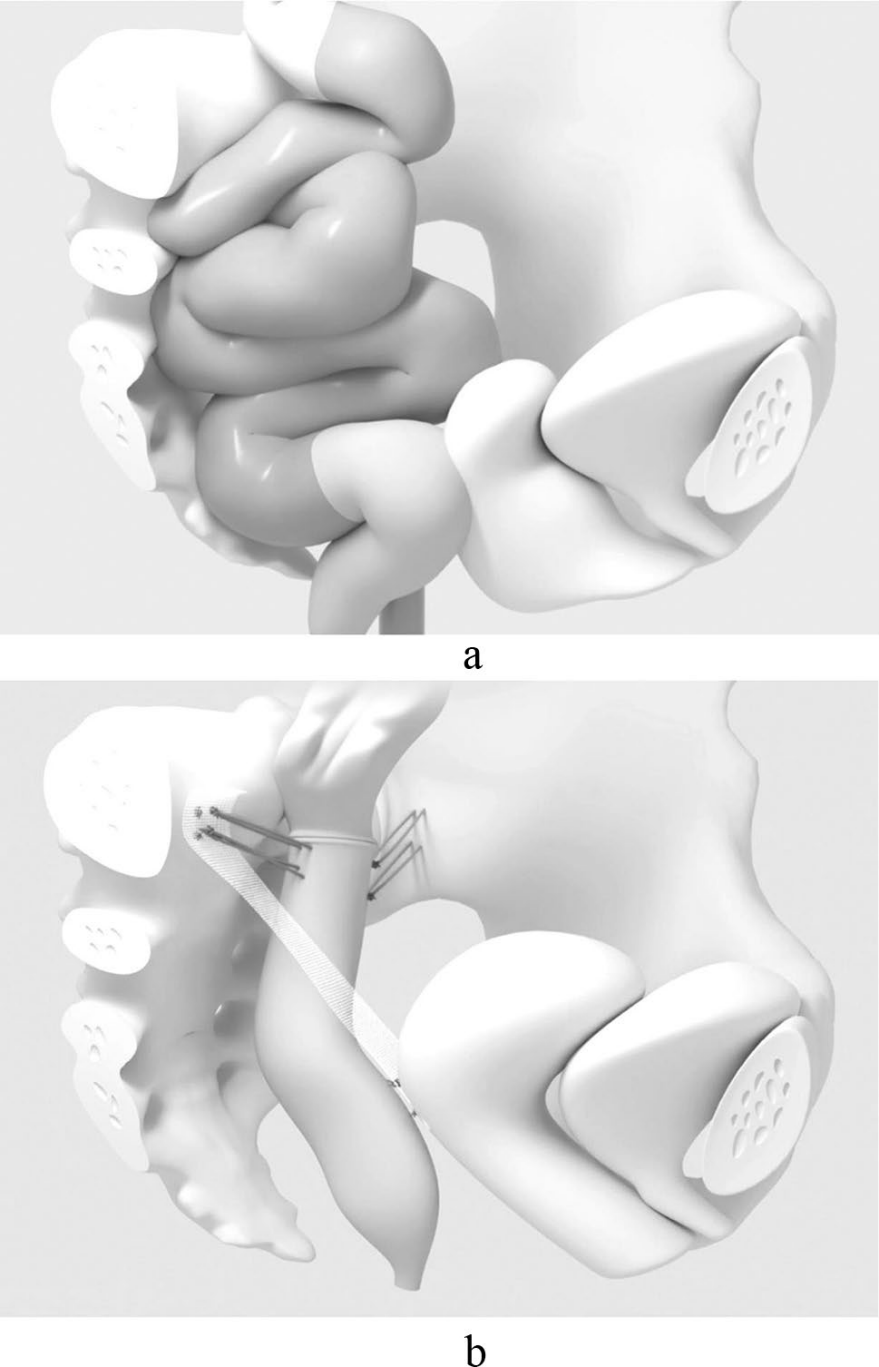
The illustration below outlines the surgical procedure in case of a female patient with ODS and POP. (**A**): Colon and rectum with a pelvic organ prolapse before surgery. The dark gray structure represents the elongated rectosigmoid. (**B**): After colorectal resection with anastomosis, a suture rectopexy attaches the rectum to both sides of the pelvis. The apical fixation of the middle compartment shows a uterus-preserving technique with a mesh fixing the dorsal part of the cervix to the promontory [22, Copyright © 2024 Facts, Views & Vision].

The procedure starts with the preparation the elongated rectosigmoid, as marked in dark grey in the illustration. By opening the retrorectal Waldeyer’s space and the Denonvilliers’ fascia or the rectovaginal space down to the pelvic floor allows for a complete mobilization of the rectum to resolve the rectocele or intussusception. Subsequently, the mesentery of the elongated rectosigmoid is dissected and after an anterior resection of the rectum and sigmoid colon, bowel continuity is restored via an end-to-end stapled anastomosis (Endoscopic Curved Intraluminal Stapler, Johnson & Johnson Medical GmbH, Norderstedt, Germany). Thereafter the rectum is fixed with suture rectopexy using a non-resorbable barbed suture to the longitudinal ligament of the promontory preferably to the right side. In female patients with POP an additional apical fixation of the middle pelvic organ compartment (uterus, cervix, or vagina) was performed as a previously described^22,23^ and illustrated in Fig. 2.

### Surgical outcome parameters

Surgical outcome parameters included the surgical operating time, hospital stay duration, and postoperative morbidity and mortality determined using the Clavien–Dindo classification (CDC)^24^. The CDC classifies the severity of complications from mild (CDC 1 and CDC 2), more severe complications that may require intervention under regional/local (CDC 3a) or general anesthesia (CDC 3b), life threatening complications with single (CDC 4a) or multiorgan failure (4b), and death (CDC 5). In general, complications classified from CDC I to CDC IIIa are considered minor, whereas those from CDC IIIb to CDC IVb are considered major complications.

### Six-month follow-up after surgery

In our center, all patients with ODS are routinely reassessed 6 months after surgery. Based on our clinical experience, the patient is considered to have an improvement in there’s a reduction of more than 3 points in the bowel function score and a strong improvement with a reduction of more than 6 points compared to the preoperative results. Similarly, deterioration and major deterioration were assumed at an increase of more than 3 or 6 points from those before surgery, respectively.

### Data management and statistical analysis

The necessary clinical data were collected preoperatively and at the 6-month postsurgical follow-up. All scores were documented on paper and transferred to a data bank. Data were analyzed using the SPSS statistical package, version 29.0.0.1. (IBM Corp., Armonk, NY, USA). Quantitative variables are presented as the means (± standard deviation) and were compared using the Kruskal–Wallis H test and Mann–Whitney U test. Qualitative variables are presented as counts, percentages, medians, and interquartile ranges and were compared using Fisher’s exact test. A two-sided p value of < 0.05 was considered to indicate statistical significance. Multiple testing was adjusted using the Bonferroni correction. To calculate the relationship between mental health and functional outcome, a two-factor analysis of variance for Friedman ranks was performed. The Kruskal–Wallis test was conducted for independent subgroups and adjusted using the Bonferroni correction. The chi-square test was conducted to compare nominal data in cross tables, which was further used in cases of statistical significance. Extreme and spike values were removed from the boxplots for better visibility.

## Results

The study included 108 consecutive patients who underwent surgery for ODS and completed the 6-month follow-up.

### Patients’ characteristics

For the 108 patients (94 [87%] women and 14 [13%] men) with complete follow-up data, the parameters of age, ASA score, and BMI (kg/m^2^) were summarized and stratified according to sex (Table 1). The median age of the patients was 57 years (23–86 years). On average, women were older than men (p = 0.032). The details are listed in Table 1.

**Table 1:** Characteristics of patients included in the analysis.

Patients’ characteristics				
	All patients (n = 108)	Female (n = 94)	Male (n = 14)	p
Age (years), median (range)	57 (23–86)	58(25–86)	38(23–84)	0.032
ASA, n (%)				0.052
1	7(6.5%)	4(4.3%)	3(21.4%)	
2	56 (51.9%)	50(53.2%)	6(42.9%)	
3	45 (41.7%)	40(42.6%)	5(35.7%)	
BMI (kg/m^2^), median (range)	24 (16–39)	24(18–39)	21(16–24)	0.002

### Preoperative clinical and mental health scores and correlations between scores

#### Altomare score

**Fig. 3:**
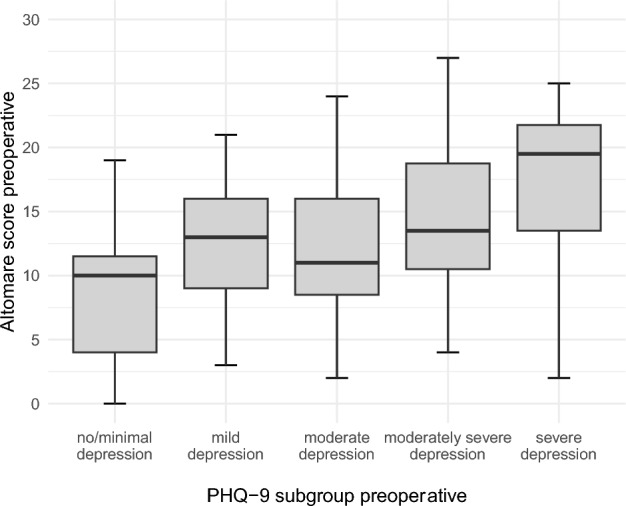
Distribution of preoperative Altomare score by preoperative PHQ-9 subgroup as illustrated in boxplots.

The preoperative Altomare score averaged 12 points (0–28 of 30 points maximum) and did not differ between sexes (p = 0.092). The Altomare score differed between the PHQ-9 subgroups, with increasing values correlating with more severe depressive symptoms (Fig. 3). No difference was observed with the GAD-7 subgroups.

#### Rectal toxicity score

**Fig. 4:**
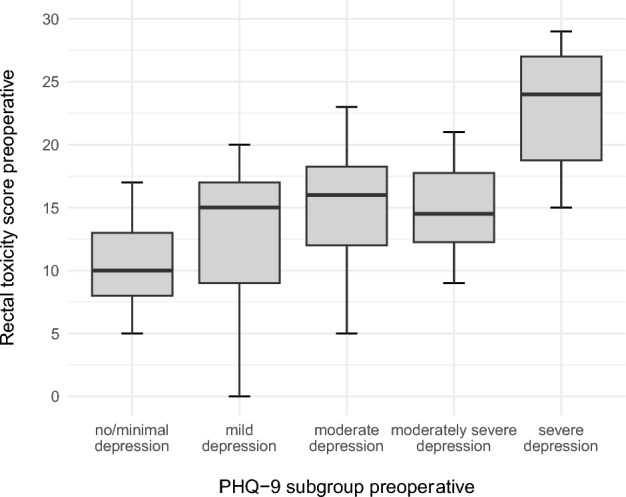
Distribution of rectal toxicity scores by PHQ-9 subgroup as illustrated in boxplots.

The preoperative rectal toxicity score was 14 points (0–29 of 32 points maximum), with no difference between female and male patients (p = 0.392). The more severe the depression, the higher the score (Fig. 4A; p = 0.204). No difference was observed with the GAD-7 subgroups, except for severe anxiety (Fig. 4B).

#### Wexner incontinence score

The Wexner incontinence score (WIS) averaged 8 points (0–20) and did not differ between sexes (p = 0.582). The score peaked with severe depression and showed no significant variation with the GAD-7 subgroups.

#### Mental health scores

The PHQ-9 score was 9 points (0–27) before surgery and 82.5% of the patients (men: 100%, women: 79.8%) had at least mild depressive symptoms (PHQ-9 score ≥ 5). Severe depressive symptoms were reported in 14.9%.

Between the PHQ-9 subgroups 19 patients had no or minimal depressive symptoms, and 45, 28, 10, and 6 patients described mild, moderate, moderately severe, and severe depression symptoms, respectively.

The distribution of the PHQ-9 score significantly differed between the sexes (p = 0.006). Men exhibited moderate (men: 42.9%, women: 23.4%) and severe (men: 28.5%, women: 12.5%) depressive symptoms more frequently than women did.

**Fig. 5:**
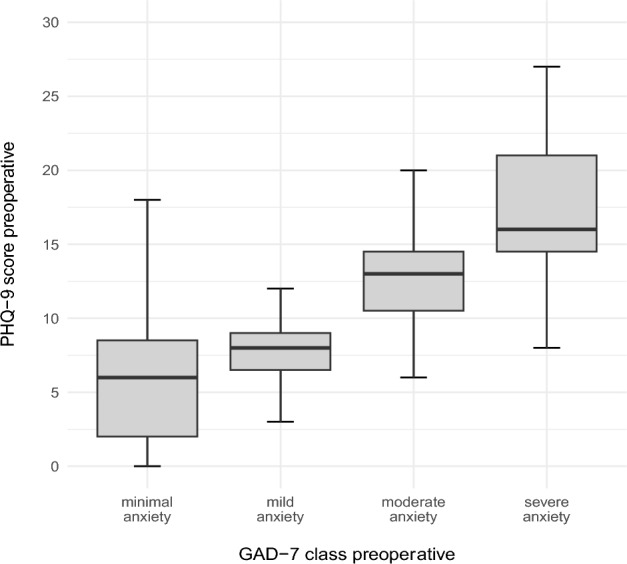
The average PHQ-9 score correlated with severity in the GAD-7 subgroups as illustrated in boxplots.

The patients in the subgroup with moderately severe depression were significantly younger (median age: 40.5 years) than those in the other subgroups were (p = 0.013). The preoperative PHQ-9 score increased with rising severity in the GAD-7 subgroup (Fig. 5).

The preoperative GAD-7 score averaged 7 points (0–21 points of 21 points maximum) for all patients and differed significantly between the sexes (p = 0.017). Men had a higher score, with a median of 11 points (1–21), than women, with a median of 6 points (0–21).

In the GAD-7 subgroups, minimal anxiety, mild anxiety, moderate anxiety, and severe anxiety symptoms were reported by 48, 26, 23, and 11 patients, respectively. At least mild anxiety (GAD-7 score ≥ 5) experienced 55.6% of the patients (men: 71.4%, women: 53.2%) and moderate anxiety symptoms by 42.9%. Severe anxiety symptoms were reported by 10.2% of all patients (men: 21.4%, women: 8.5%).

The patients with severe anxiety symptoms were significantly younger (median age: 41 years) than those in the other subgroups (p = 0.038).

**Fig. 6:**
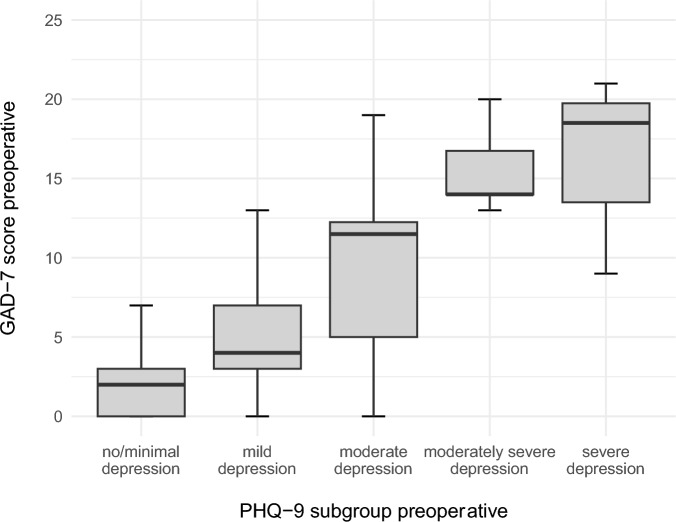
Preoperative GAD-7 scores correlated with preoperative PHQ-9 scores as illustrated in boxplots.

The GAD-7 scores increased with more severe depression scores (Fig. 6).

### Surgical outcomes

All participants underwent laparoscopic surgery with no conversion to open surgery. The median operating time was 247 min (range: 78–450 min) and differed significantly between both sexes (p < 0.001). The median postoperative hospital stay duration was 8 days (3–21 days).

In total, 12 participants (11.2%) experienced postoperative complications, eight of which were minor (CDC 1–3a). The major complications (n = 4) were postoperative ileus (n = 3) and anastomotic leakage (n = 1). No deaths occurred.

### Clinical outcome for bowel function scores 6 months after surgery

**Fig. 7:**
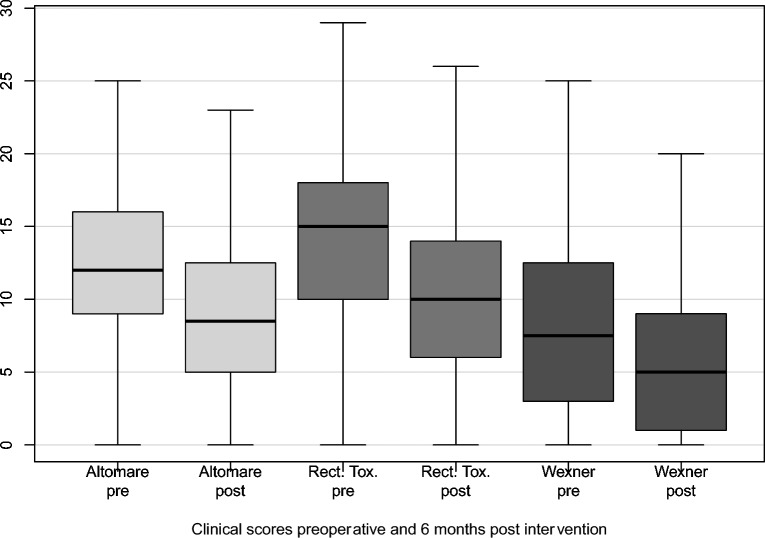
Bowel function scores preoperatively and at the 6-month follow-up were illustrated in boxplots.

A significant improvement was observed for all bowel function items collected (Fig. 7). Altomare score dropped from 12 points preoperatively) to 9 points (0–23) at 6-month follow-up (p < 0.001). The rectal toxicity score averaged (14 points) before surgery and decreased significantly to 10 points (0–27) after surgery (p < 0.001). The preoperative Wexner incontinence score (8 points) improved significantly to (6 points (0–20)) after surgery (p < 0.001).

### Correlations between preoperative mental health and postoperative bowel function outcomes

**Fig. 8:**
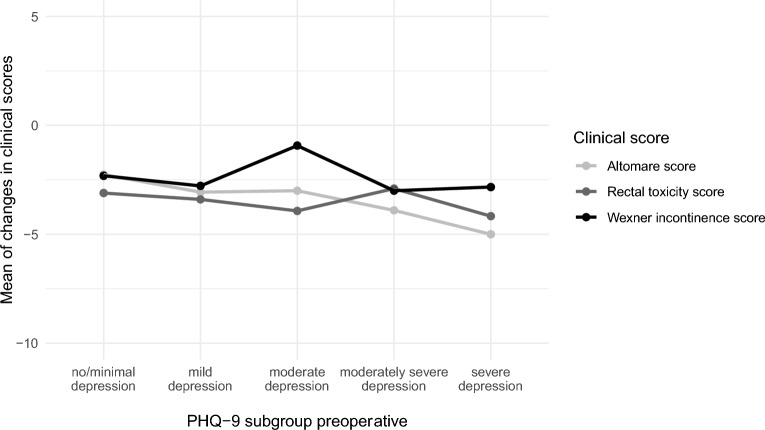
The mean changes in clinical scores after surgery (point) are linked (lines) in a graph according to the respective preoperative PHQ-9 subgroup.

The preoperative severity of depressive symptoms (PHQ-9 subgroups) had no statistically significant impact on the postoperative outcome of the bowel function scores (Altomare score: p = 0.654, rectal toxicity score; p = 0.985, and Wexner incontinence score: p = 0.618) (Fig. 8). This finding was observed similarly for the preoperative severity of anxiety symptoms (p = 0.709, p = 0.139, and p = 0.548, respectively).

### Follow-up results for mental health questionnaires

#### PHQ-9 scores after surgery

The PHQ-9 reflected postoperative improvement in depression status, with an average score of 8 points (0–24) noted at 6 months.

**Fig. 9:**
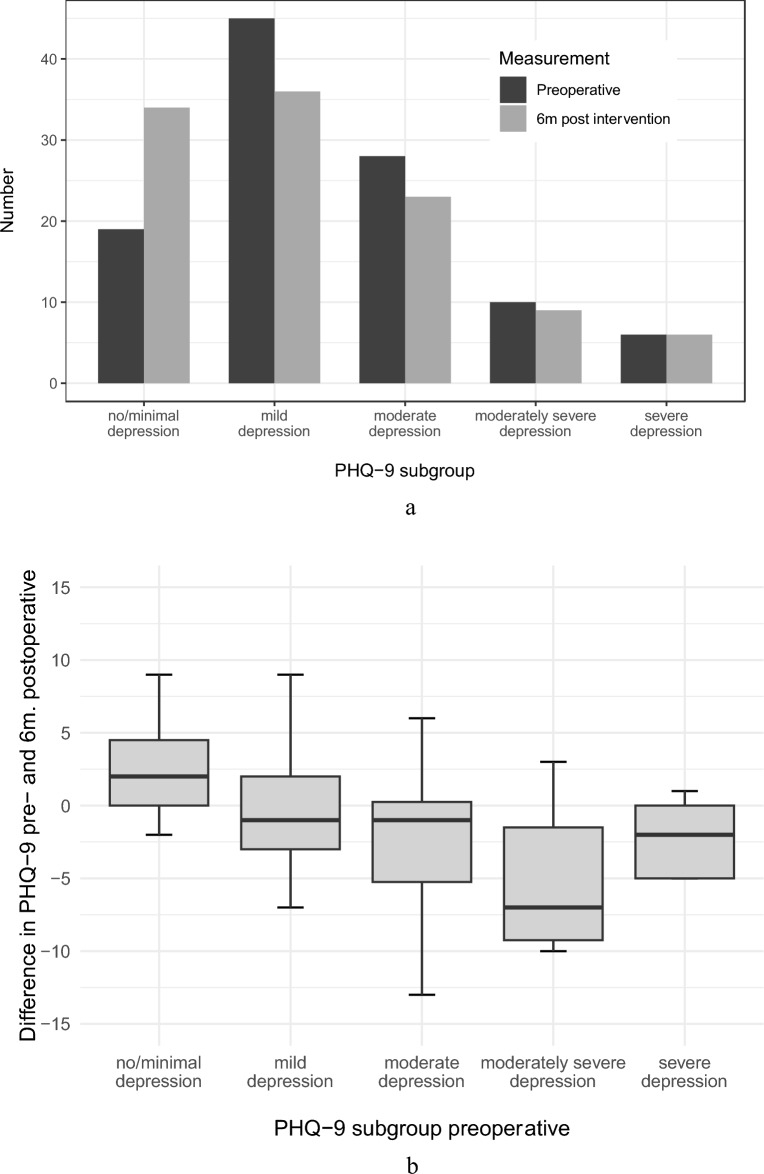
Absolute number of patients in the PHQ-9 subgroups shown in columns before (black) and 6 months after surgery (grey) (**A**). Changes in the PHQ-9 score after 6 months in correlation with the preoperative PHQ-9 subgroup illustrated in boxplots (**B**).

After surgery, the group of patients with least mild depressive symptoms was reduced to 67.6%; the changes in the total cohort (p = 0.075) and the difference between sexes was not significant (p = 0.051). The distribution between subgroups in absolute numbers is illustrated in a block diagram (Fig. 9A).

The changes in PHQ-9 scores after surgery correlated with the initial preoperative PHQ-9 scores with the most significant improvement observed in the moderately severe depressive subgroup (Fig. 9 B). Improved PHQ-9 scores were observed significantly in the preoperative GAD-7 subgroup with moderate anxiety symptoms as visualized in boxplots (Fig. 9B).

#### GAD-7 score after surgery

The GAD-7 scores improved slightly after surgery (7 points (0–21); p = 0.51) with a statistically significant difference between sexes (p = 0.07).

**Fig. 10:**
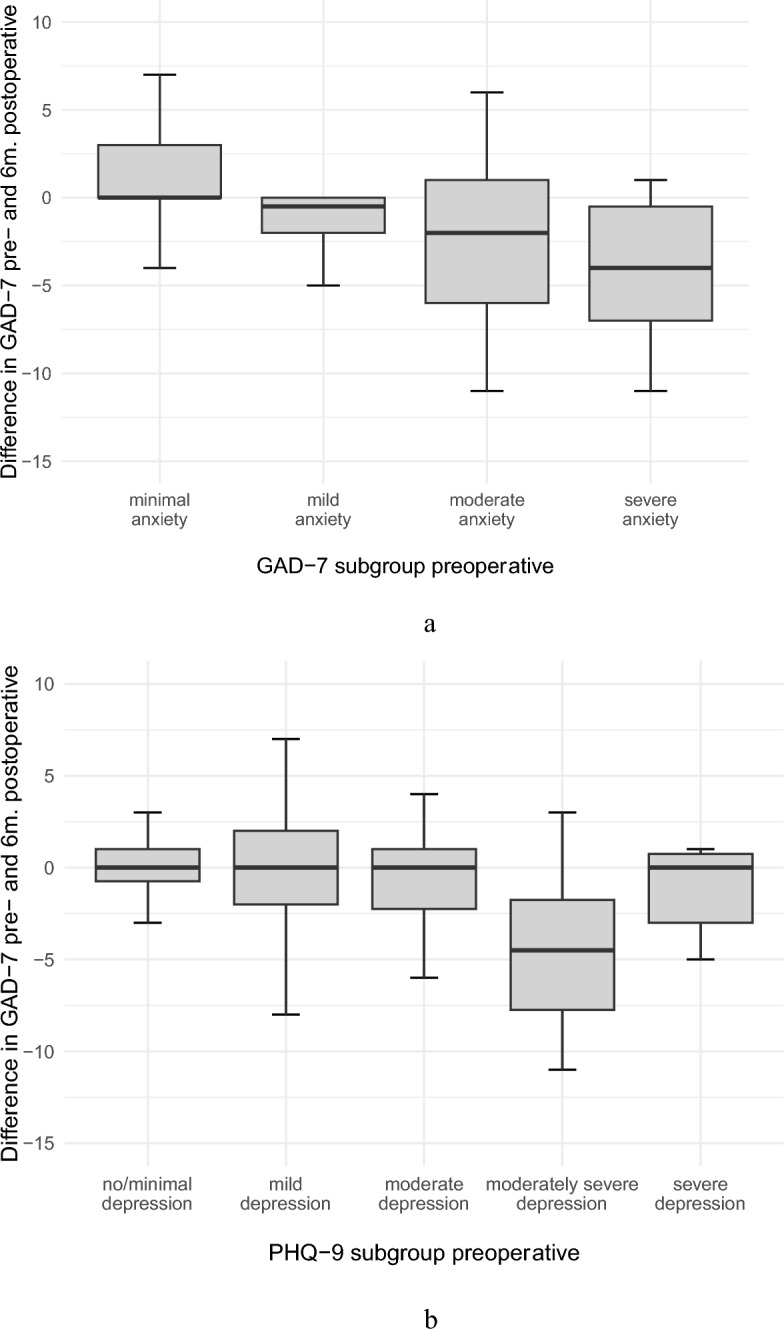
Changes in the GAD-7 scores after 6 months in the preoperative GAD-7 (**A**) and PHQ-9 subgroups (**B**) as illustrated in boxplots.

The changes in GAD-7 scores in correlation with the preoperative values revealed pronounced symptom improvement in the moderate anxiety and severe anxiety subgroups (Fig. 10A). The changes in postoperative GAD-7 scores improved significantly in the initial moderately severe PHQ-9 subgroup (p = 0.028) (Fig. 10B).

### Correlations between mental health outcomes and clinical scores

The clinical outcome scores were rated according to their changes at 6 months after surgery, as outlined in the Methods section, and correlated with the changes in the postoperative PHQ-9 and GAD-7 scores.

#### PHQ-9 and Altomare scores

**Fig. 11:**
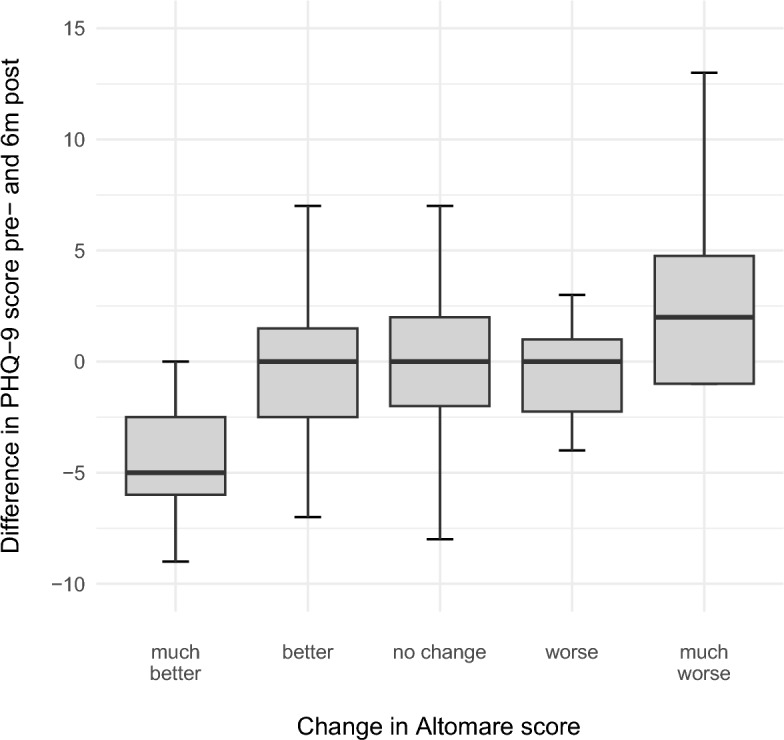
Changes in PHQ-9 scores at 6 months after surgery according to postoperative Altomare subgroup shown in Boxplots.

The changes in PHQ-9 scores differed significantly across the categories of Altomare scores (p = 0.016). The most significant improvement in PHQ-9 scores was observed in the subgroup exhibiting major improvement in Altomare scores (p = 0.019) (Fig. 11).

#### PHQ-9 and rectal toxicity scores

The changes in PHQ-9 scores after surgery correlated with those observed in the rectal toxicity scores, with the most prominent changes observed in the group with much better rectal toxicity results (p = 0.011).

#### PHQ-9 score and Wexner incontinence score

Changes in the PHQ-9 score after surgery were not significantly correlated with postoperative changes in the Wexner incontinence score (p = 0.074).

#### GAD-7 and clinical scores

Changes in the GAD-7 score after surgery were not significantly correlated with improvements in the Altomare score (p = 0.191), rectal toxicity score (p = 0.231), or Wexner incontinence score (p = 0.304).

## Discussion

The present study reports a high mental burden in a large cohort of patients undergoing surgery for ODS.

Overall, 82.5% of our patients showed at least mild depressive symptoms, based on the PHQ-9 scores. This highlights a considerably greater depressive burden in patients with ODS than in patients suffering from other somatic diseases, such as those with rheumatism (38.8%), for example^25^.

Anxiety disorders are more common in patients with somatic diseases than in the general population, with a prevalence ranging from 2.5% to 55%^26^. In our study, 55.6% of the ODS patients had at least mild anxiety, which is even above the upper limit of the known prevalence range.

These results are consistent with studies that have associated somatic comorbidities with depressive and anxiety disorders with a high odds ratio^27^. The symptom complex that precedes and accompanies ODS, i.e. constipation and irritable bowel syndrome, is associated with a significantly greater prevalence of anxiety and depression than in healthy controls^28,29^ and mental comorbidities impair the quality of life of these patients^30,31^. And interestingly, patients in our study with higher PHQ-9 and GAD-7 scores were significantly younger than those in the rest of the study population.

ODS is known to be significantly more prevalent in women^16,32^, which was confirmed in the present study with the majority (almost 90%) of the included patients being women. Interestingly, significant differences were observed between the female and male populations in our study. Male patients were significantly younger and had a lower BMI, which explains the lower number of comorbidities in the male patients compared to the female patients. However, the male cohort, had a significantly greater psychological distress at diagnosis, with higher scores for both depression and anxiety symptoms than the female cohort. This may be related to the lower likelihood of men reporting bowel function symptoms to their physicians, which leads them to feel more isolated. In contrast, women routinely consult their gynecologists, who are increasingly aware of their comorbidities, and can discuss their POP-related symptoms, which are often associated with functional bowel disorders^6,16,32^.

The severity of the depressive symptoms correlated with the clinical severity of ODS and bowel dysfunction symptoms in study population, suggesting that more severe somatic ODS symptoms cause a greater mental burden in affected patients. Interestingly, this was not observed for incontinence symptoms and the correlation between the clinical scores and the anxiety score subclasses did not reach statistical significance.

The surgical intervention showed excellent operative results and a low postoperative morbidity. The significantly longer operative time for female patients was due to the simultaneous pelvic floor reconstruction.

At the 6-month follow-up, all clinical bowel function scores were significantly improved. Depression symptoms also improved after surgery, with only 67.6% of all patients still being above the threshold for at least mild depressive symptoms. The incidence of moderately severe (21.3% of all patients) and severe (13.9% of all patients) depressive symptoms also decreased after surgery. However, no significant improvement in anxiety symptoms was observed. While the improvement of depressive symptoms correlated with the improvement of the somatic burden and seemed to be a more transient response related to the clinical burden of ODS in this cohort, the anxiety scores were more stable and suggest a possible personality trait. Future studies need to take a more nuanced view of anxiety symptoms and possible underlying different forms of anxiety disorders, distinguishing state anxiety from trait anxiety, the latter being much less likely to change on an individual patient basis, with evidence of a neuroanatomical and functional distinction between state and trait anxiety^33^. The fact that our patients did not show significant changes in anxiety symptoms suggests the predominance of the latter in our cohort and implies a possible difference in help-seeking behavior between these two groups^34^.

Next, we determined whether the improvement in mental health after surgery was a result of the improved clinical outcome. This hypothesis was validated, as the PHQ-9 and GAD-7 scores were significantly lower in the subgroup of patients with improved or much improved clinical symptoms after surgery. In addition, patients with worse or much worse clinical outcomes did not show any improvement in their mental health status.

### Discussing the results with the literature

Few studies with small sample sizes have explicitly investigated ODS and mental health status. In our study, depressive symptoms were observed in 82.5% of our patients (with moderate to severe symptoms in 40.8%), and anxiety symptoms were observed in 55.6% of our patients (with moderate to severe symptoms in 33%). Other studies have reported high but not uniform and sometimes even different values for this group of patients.

Groenendijl et al. (2012) compared women with POP with a control group without POP and observed clinical depressive symptoms in 36% of POP patients using the self-reported Centre for Epidemiological Studies Depression Scale. Anxiety was not assessed in this study^35^.

In the study by Duca et al. (2023), 28 of the 36 ODS patients (77.8%) reported depressive symptoms (HADS > 8), and 30 of the 36 patients (83.3%) reported anxiety symptoms (GAD-7 > 7 points)^8^.

In 2022, Yiang et al. investigated the diagnosis of functional bowel disorder and the correlation of patient characteristics with clinical health, mental health, and quality of life. They found that anxiety, as measured by the GAD-7 questionnaire, was strongly correlated with defecation symptoms and physical discomfort^9^.

Our findings are consistent with those of previous studies reporting a high burden of anxiety and depression in ODS patients. However, only a few studies have addressed the mental health issues in ODS patients undergoing surgery. In addition, most surgical studies have used a transanal approach, which is no longer the current treatment of choice.

Dodi et al. (2003) were the first to follow up 14 chronically constipated female patients who underwent ODS surgery after transanal stapled rectotomy and who presented with severe complications or recurrence after surgery. Psychoneurosis, a synonym used for depression and anxiety disorders, parity, and a spastic pelvic floor were identified as risk factors for surgical failure^36^.

Pescatori et al. (2006) evaluated underlying “occult disorders” in 100 patients with conservative or surgical treatment of ODS and reported an incidence of anxiety or depression of 66%. However, they did not differentiate between the two symptom complexes, nor did they specify the instrument used to measure prevalence^37^. Their updated results for a subgroup of 80 patients undergoing tailored (transanal) surgery for ODS (2022) showed that half of the patients suffered from psychological distress, which was not further specified^38^.

In contrast, Wolff et al. (2010) reported significantly improved somatic and depression scores as well as health-related quality of life in 52 female patients after transanal stapling surgery for ODS^39^. However, the study did not present follow-up results.

In 2015, Qin et al. analyzed 19 patients after transanal stapling surgery and compared the results obtained of the Hamilton Depression Rating Scale, the Hamilton Anxiety Scale, and anorectal dynamics with findings of 9 healthy volunteers. After surgery, the short-term improvement was comparable to that of the healthy controls. However, the results returned to the elevated preoperative levels after 90 days. The authors concluded that ODS patients are prone to anorectal, mental, and psychological disorders^40^.

Since transanal stapling surgery for ODS has been abandoned over the years due to unsatisfactory long-term results^41–43^, the studies based on this surgical technique should be included in general discussion with caution. Fortunately, some recent studies have investigated the relationship between mental health status and surgical outcomes in colorectal surgery in general, as well as the effect of depression and anxiety disorders on the outcome of laparoscopic ODS surgery.

In 2022, Maroli et al. investigated the short-term effect of preoperative anxiety on postoperative complications in 65 (mostly male) patients undergoing colorectal surgery. Their results associated female sex, depression, and stress with higher preoperative anxiety levels (HADS > 11) and identified anxiety as an independent risk factor for postoperative complications within 30 days of surgery^44^.

In a long-term analysis after ventral mesh-rectopexy for rectal prolapse, Marra et al. (2023) reported that patients with anxiety and depression benefited significantly less from surgery and had a higher rate of recurrence after surgery than did mentally healthy patients. The authors recommended that anxiety and depression status be considered in surgical decision-making^10^. However, Cao et al. (2022) investigated the long-term functional outcome after ventral mesh-rectopexy for ODS in patients with or without anxiety/depression and reported no impaired functional outcome for the affected patients^29^. These majority of these studies have suggested the role of mental health as an independent risk factor for surgery and surgical outcome in functional bowel surgery without defining its exact role and implications for the medical care of ODS patients.

This study found that clinical symptom scores and preoperative mental distress were strongly correlated. The short- and long-term outcomes for clinical scores and the mental distress symptom complex improved significantly in our patients after surgery, which contrasts with the results of most of the aforementioned studies.

In addition, although greater preoperative depression and anxiety symptoms were correlated with clinical symptoms and vice versa, they did not affect short-term surgical results or 6-month follow-up outcomes. Rather, improvement in mental health relief was directly related to clinical improvement and was not observed in patients who did not experience improvement in their clinical symptoms. This direct correlation between improved clinical outcomes and better mental health scores reinforces the importances of an individualized approach for this patient population.

Additional support for our findings comes from the first randomized controlled trial investigating laparoscopic ventral mesh rectopexy (LVMR) in patients with internal rectal prolapse. Conducted in the UK across six institutions using a stepped-wedge design, the study demonstrated significant improvements in patient-reported outcomes at 24 weeks, remaining significant at 72 weeks. However, the sample size was limited due to a national moratorium on mesh surgeries (n = 28, drop out n = 9) and no significant changes were observed in generalized anxiety or depression scores (GAD-7, PHQ-9). The authors concluded that surgical intervention can meaningfully impact illness behavior and disease burden, even in the absence of measurable changes in psychological traits such as anxiety. The study thus reinforces the psychosomatic dimension of surgical outcomes in ODS and supports the need for integrative treatment approaches ^45^.

The individualized diagnosis and treatment of ODS is quite challenging and requires a comprehensive approach that also considers the mental and psychological distress of affected patients. We propose a thorough multidisciplinary diagnostic work-up, consensual therapeutic decision making by an interdisciplinary medical board, a tailored laparoscopic surgical approach if indicated in an interdisciplinary team, if necessary. Based on the results from the psychosomatic self-report questionnaires, we recommend that psychosomatic screening should take place as early as the patient’s first presentation to a tertiary pelvic floor center and definitely prior to a planned surgical intervention. This may include referral to a psychosomatic consultation service in cases where psychological distress is suspected. Given the often long-standing and individually variable course of symptoms, the point of first specialized contact should serve as the earliest feasible opportunity for such an evaluation.

### Strengths and limitations

To our knowledge, the present study is the largest analysis of the relationship between mental health and the clinical symptom severity in patients with ODS undergoing surgery. Furthermore, this study compares depression and anxiety symptoms in the included patients before and after surgery. The large number of male patients in our cohort strengthens the robustness of our conclusions.

Despite these strengths, our study also has several limitations. First, the results were obtained exclusively from self-report questionnaires and lacked information on mental health conditions diagnosed before or after ODS onset. Furthermore, we assessed only subjective symptom scores without systematically collecting diagnostic criteria for possible pre-existing mental illnesses.

In addition, the absence of a control group (i.e. patients who did not undergo surgery or were managed differently) makes it challenging to attribute the observed positive changes exclusively to the surgical procedure. Being treated at a specialized center, where the importance of the patient’s symptoms may be specifically emphasized, might have enhance the patients’ responsiveness to treatment due to several factors, such as heightened patient expectations, staff expertise, or specialized protocols.

Moreover, no data were collected on whether the included patients were receiving psychotherapy, medication, or other mental health treatments, which could potentially influence changes in pre-post intervention scores.

To address these limitations, we will further investigate mental distress and psychological comorbidity in patients with ODS in collaboration with the psychosomatic department. Our first step will be a prospective, randomized study in which we compare findings from a structured psychosomatic interview conducted at the initial patient presentation with results obtained exclusively from self-report questionnaires.

## Conclusions

Patients with ODS are highly burdened by depression and anxiety symptoms, which have an enormous impact on their quality of life. The severity of the clinical symptoms directly correlates with their mental distress and vice versa.

Based on the results obtained, we propose that these patients be treated by a multiprofessional team with an individualized approach. Since a surgical intervention is available for a large subgroup of patients with ODS and patients’ mental well-being improves with good functional outcomes, these patients should be offered this treatment option at an early stage. Early and routine screening for and treatment of mental disorders should be offered in addition to consistent conservative and surgical intervention and will be critical to the effective management of these patients. Therefore, we recommend that all patients presenting at specialized pelvic floor centers—such as our interdisciplinary tertiary Pelvic Floor Center—undergo psychosomatic screening, ideally at their first presentation and definitely prior to any surgical intervention. Future research should address the mental health of ODS patients using self-report questionnaires as well as structured clinical interviews. Other possible mental disorders such as somatoform or somatization disorders, persistent somatoform pain disorders, or obsessive–compulsive disorders should also be investigated.

## Data Availability

The data that support the findings of this study are not openly available due to reasons of sensitivity and are available from the corresponding author upon reasonable request.
